# Greenhouse gas emissions of a large, academic outpatient orthopedic center in the United States

**DOI:** 10.3389/frhs.2025.1675827

**Published:** 2025-10-20

**Authors:** Anna M. Jett, Venkat Kothandaraman, Esther Bobbin, Seth Sheldon, Lisa M. Colosi, Matthew J. Meyer

**Affiliations:** ^1^ Office for Sustainability, University of Virginia, Charlottesville, VA, United States; ^2^ Department of Anesthesiology, Northwestern University, Chicago, IL, United States; ^3^ Rho Impact, Charlottesville, VA, United States; ^4^ School of Engineering, University of Virginia, Charlottesville, VA, United States; ^5^ School of Medicine, University of Virginia, Charlottesville, VA, United States; ^6^ Medical Director of Sustainability, UVA Health, Charlottesville, VA, United States

**Keywords:** pollution, waste, healthcare, orthopedics, greenhouse gas, emissions, quality improvement, community health

## Abstract

**Introduction:**

Hospitals and health systems create pollution as a byproduct of their work improving people's personal health. Pollution can harm human health. As part of a broad effort to comprehensively quantify a health system's pollution, we started with one group of pollutants, greenhouse gases, at a freestanding outpatient orthopedic center (OC).

**Methods:**

OC has clinic rooms, imaging, administrative offices, and a small ambulatory surgery center. It was newly constructed and received LEED Silver certification in 2022. The Greenhouse Gas Protocol was used to categorize emissions into Scope 1 (direct), Scope 2 (indirect from purchased energy), and Scope 3 (supply and value chain) emissions for fiscal year 2023.

**Results:**

OC's total annual emissions were 11,049 metric tons of carbon dioxide equivalent (MTCO2e), with 2% from Scope 1, 17% from Scope 2, and 81% from Scope 3. Most Scope 3 emissions came from just three categories: patient transportation (52% of Scope 3 emissions), purchased goods and services (20%), and employee commuting (12%).

**Discussion:**

This initial study highlights the significant contribution of Scope 3 emissions to an outpatient center's greenhouse gas footprint. It specifically identifies patient travel as a major contributor to emissions; this is particularly important since patient travel is not always included in Greenhouse Gas Protocol healthcare assessments and patient travel can be mitigated in some circumstances by utilizing telemedicine. The emissions distribution across scopes is similar to other international hospitals, indicating generalizability, though the high proportion of patient travel emissions is unique to this outpatient-focused facility.

## 1 Introduction

Human health is affected by environmental health. People providing products and services for healthcare should be conscientious of their industry's contribution to environmental harm. One method of monitoring healthcare's consumption of resources and contribution to pollution is accounting for greenhouse gas (GHG) emissions.

Globally, the healthcare sector is responsible for 4.4% of total GHG emissions (1). In the United States, it is responsible for 8.5% of national emissions (2). Using the Greenhouse Gas Protocol (3) to categorize emissions from the entire US healthcare sector: 7% were directly emitted by healthcare industry buildings and operations (Scope 1), 11% were associated with purchased energy, such as electricity and steam (Scope 2), and 82% were associated with the services and products produced and consumed (Scope 3) by the healthcare sector (2).

Scope 1 and Scope 2 emissions are more easily quantified as they are either directly controlled by an organization and/or can be monitored through metering or billing. Scope 3 emissions are more complicated and typically require data from a variety of different sources to assess the emissions generated from the production and consumption of products and services. Due to the large contribution of Scope 3 emissions to total emissions in healthcare, it is important that these emissions are well characterized to understand how to most effectively utilize healthcare resources to reduce pollution while maintaining high-quality care.

In the hospital setting, life cycle analyses have been published and aggregated for a variety of different healthcare related items: medications, devices, supplies, clinical procedures and processes, even entire clinical wards (4). However, across the world there have been few published assessments of the GHG emissions of entire hospitals in peer-reviewed journals (5–11). UVA Health is aligned with the University of Virginia, which has a goal of Scope 1 and Scope 2 carbon emission neutrality by 2030. Considering the proportion of Scope 3 emissions in healthcare, we want to assess UVA Health's total GHG emissions, inclusive of Scope 3. To do this, we initiated a process to account for Scope 1, Scope 2 and Scope 3 emissions inside of our health system with a pilot study that identified, calculated, and analyzed emissions at a large, representative clinical building using the Greenhouse Gas Protocol (3).

## 2 Methods

The building selected for the pilot was a freestanding orthopedic center associated with UVA Health (Charlottesville, VA, USA) called Orthopedic Center Ivy Road (OC). It has discrete physical, supply, and energy boundaries from the larger medical center and health system, while containing many functions of larger hospitals. This building was opened in February 2022 and received LEED (Leadership in Energy and Environmental Design) Silver certification. It has 195,000 square feet (18,116 m^2^), 90 exam rooms, two operating rooms (with the potential to add more), surgical services, medical imaging, prosthetics manufacturing in a fabrication lab, a retail and institutional pharmacy, and a cafeteria.

A modified operational boundary was applied to OC for the purposes of the GHG inventory. OC is a freestanding center associated with UVA Health's orthopedic clinical care. The GHG inventory included emissions resulting from all operations occurring within the property limits of OC.

One year of data was used for all sources (Fiscal Year 2023: July 1, 2022 to June 30, 2023), and the center had over 300 full-time equivalent employees (FTE) onsite during the data collection. As FTE is a unit of measure that corresponds the collective hours of all full- and part-time employees to the equivalent number of full-time employees who could work those hours, it does not account for the impact of patients or visitors onsite. IRB approval was waived: this analysis was performed as part of a quality initiative project and is independent of specific patient data.

### 2.1 Greenhouse gas protocol

The Greenhouse Gas Protocol (3), a widely recognized standard for measuring and managing GHG emissions, has established a framework for categorizing emissions into three distinct scopes to comprehensively understand their carbon footprint and identify areas for emissions reduction.

Scope 1 emissions are direct emissions that occur from sources owned or controlled by an institution (3). These emissions typically arise from on-site combustion of fossil fuels in buildings, vehicles, and equipment. At OC, on-site combustion emissions stem from a natural gas boiler and a gasoline-powered vehicle. Natural gas consumption is measured by a utility meter, while mobile fuel consumption is estimated based on the distance traveled by the vehicle, which is tracked through travel logs, and the average miles per gallon for the vehicle's make and model.

Scope 1 emissions also arise from the release of purchased gases, including waste anesthetic gases when they are vented to the atmosphere. The anesthetic gases used in OC's operating rooms are sevoflurane and nitrous oxide (e-cylinders). The volume of each anesthetic gas purchased during fiscal year 2023 was obtained from procurement data.

Scope 2 emissions are indirect emissions associated with the generation of electricity, steam, heating, and cooling purchased by an institution (3). Consumption of purchased energy at OC is measured through utility meters for electricity, hot water and chilled water.

Scope 3 emissions are indirect emissions that occur in the value and supply chain of an institution, but are not directly owned or controlled by the institution (3). There are fifteen categories of Scope 3 emissions, though not all of these categories will be relevant to all organizations (see Supplementary Table S1 for explanation of category relevance or irrelevance to OC; see Supplementary Table S2 for methodology details for the relevant categories' emissions calculations).

While patient travel is not explicitly listed in the Greenhouse Gas protocol, emissions from customer travel can be included in Category 9: Downstream transportation and distribution (3). For this study, patient travel was deemed the equivalent in health care to customer travel for retail facilities, and patient travel emissions were included in Category 9.

### 2.2 Hybrid approach

This study employed a hybrid approach to measure Scope 3 GHG emissions, utilizing both activity and financial data. Each relevant category of Scope 3 emissions used either financial or activity data (see Table 1). This method is consistent with emerging best practices, which recognize a hybrid approach as a combination of the benefits of activity- and expenditure-based methods (12). Activity data, which refers to a quantitative measure of an activity that results in GHG emissions (3), tends to produce more accurate results, but is often more complex to obtain than financial data. Activity data was collected to measure Scope 1 and 2 emissions. For Scope 3 emissions, activity data was collected when feasible, and where activity data collection was not practical, financial data was utilized (see Table 1).

**Table 1:** Identification of methodologies and emission factors databases for scope 3 categories relevant to the pilot GHG assessment of a freestanding orthopedic center.

Scope 3 category		Method	Data approach	Emission factors
1	Purchased goods and services	Spend-based method	Financial	Provided in PGH Health Care Emissions Impact Calculator V1.3 (14)
2	Capital goods	Spend-based method	Financial	Provided in PGH Health Care Emissions Impact Calculator V1.3 (14)
3	Fuel and energy related activities	Average-data method	Activity	Provided in PGH Health Care Emissions Impact Calculator V1.3 (14)
4	Upstream transportation and distribution	Distance-based method	Activity	EPA's 2023 GHG Emission Factors Hub (Table 8) (12)
5	Waste generated in operations	Waste-type-specific method	Activity	EPA's 2023 GHG Emission Factors Hub (Table 8) (12)
6	Business travel	Air travel: Distance-based method	Activity	Provided in ICAO Carbon Emissions Calculator (15)
		Lodging: Spend-based method	Financial	EPA's Supply Chain GHG Emission Factors (v1.2) (13)
7	Employee commuting	Distance-based method	Activity	EPA's 2023 GHG Emission Factors Hub (Table 10) (12)
9	Downstream transportation and distribution	Average-data method	Activity	EPA's 2023 GHG Emission Factors Hub (Table 8) (12)

EPA, Environmental Protection Agency; PGH, Practice Greenhealth; ICAO, International Civil Aviation Organization.

### 2.3 Metric tons of carbon dioxide equivalent

GHG emissions are summarized as metric tons of carbon dioxide equivalent (MTCO2e). Their GHG footprint is a summation of carbon dioxide, nitrous oxide, and methane emissions. These values came from public databases and calculators like the US Environmental Protection Agency's GHG Emission Factors Hub (13), US Environmental Protection Agency's Supply Chain Greenhouse Gas Emission Factors V1.2 (14), Practice Greenhealth's Health Care Emissions Impact Calculator V1.3 (15), and ICAO Carbon Emissions Calculator (16). They are linked to specific Scope 3 categories (see Table 1).

## 3 Results

**Figure 1:**
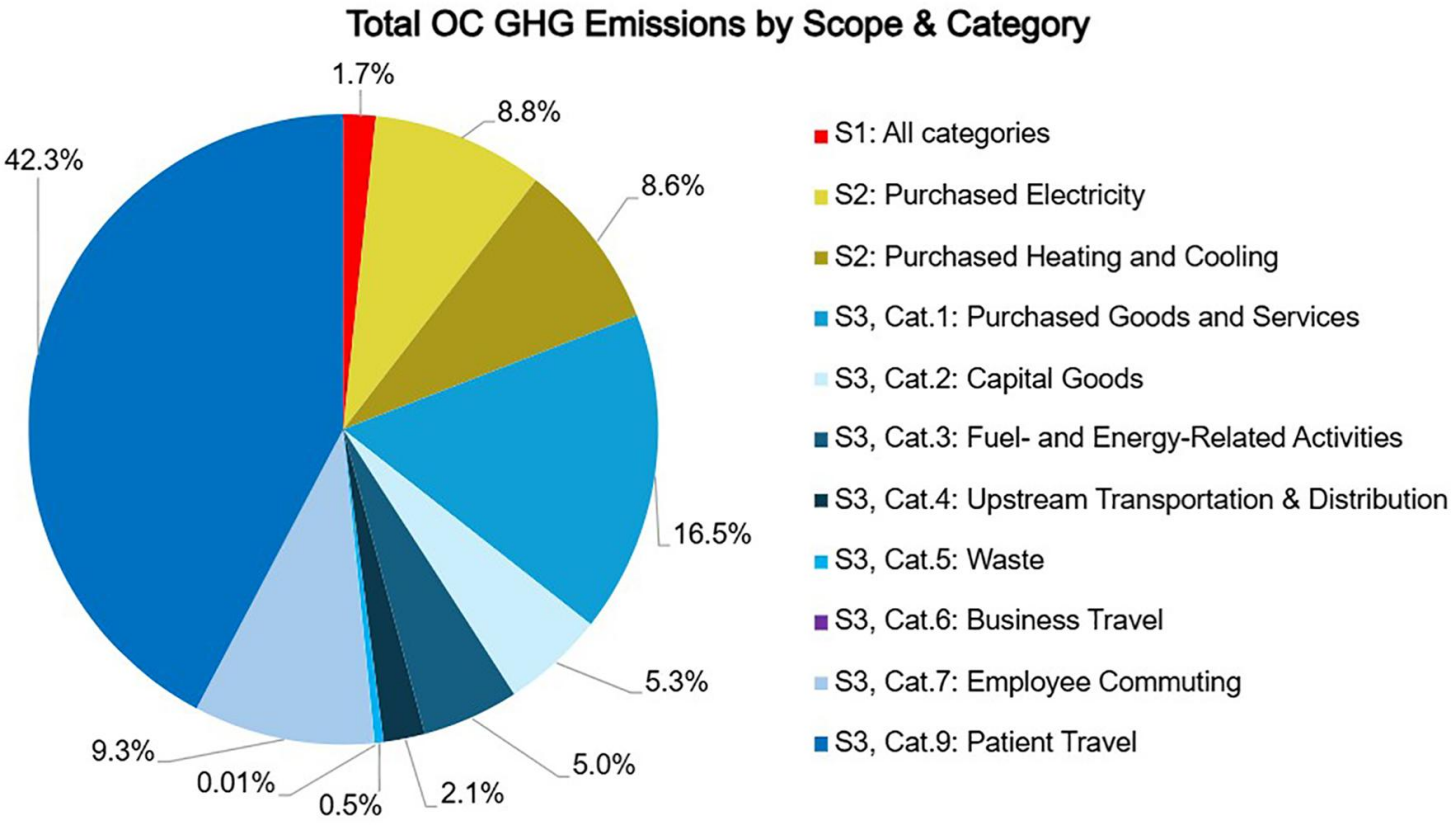
Source of total GHG emissions of a freestanding orthopedic clinic by scope and category. OC: orthopedic center.

Total emissions during fiscal year 2023 from the freestanding orthopedic center were 11,049 MTCO2e: 2% (183 MTCO2e) of these emissions came from Scope 1, 17% (1,929 MTCO2e) from Scope 2, and 81% (8,937 MTCO2e) from Scope 3 (see Figure 1; Table 2). Total emissions per FTE were 33.2 MTCO2e per FTE per year. Total emissions per square meter were 0.6 MTCO2e per square meter per year.

**Table 2:** Total emissions identified in pilot GHG assessment of a freestanding orthopedic center.

Emission category	Calculation method	July 2022–June 2023	
		Emissions (MTCO2e)	Ratio (%)
Scope 1 Direct emissions, including those from internal fuel combustion and industrial processes	Activity data	183	1.7%
Scope 2 Indirect emissions from consumption of purchased electricity, heat, or steam	Activity data	1,929	17.4%
Scope 3 Other indirect emissions	Varies by category	8,937	80.9%
Scope 1, 2, 3 Total		**11,049**	100%
Scope 1 Components			
Stationary combustion	Activity data	173	94.7%
Mobile sources	Activity data	1	0.4%
Fugitive emissions	Activity data	0	0.0%
Purchased gases	Activity data	9	4.9%
Scope 1 Subtotal		**183**	100%
Scope 2 Components			
Purchased electricity	Activity data	974	50.5%
Purchased heating and cooling	Activity data	954	49.5%
Scope 2 Subtotal		**1,929**	100%
Scope 3 Categories			
Category 1: Purchased goods and services	Spend-based	1,823	20.4%
Category 2: Capital goods	Spend-based	581	6.5%
Category 3: Fuel- and energy-related activities	Activity data	547	6.1%
Category 4: Upstream transportation and distribution	Activity data	235	2.6%
Category 5: Waste generated in operations	Activity data	50	0.6%
Category 6: Business travel	Spend-based	1	0.0%
Category 7: Employee commuting	Activity data	1,027	11.5%
Category 9: Downstream transportation and distribution	Activity data	4,672	52.3%
Scope 3 Subtotal		**8,937**	100%

Scope 1 emissions were primarily from a single natural gas boiler (173 MTCO2e; 95%). The remaining emissions stemmed from anesthetic gases and one gasoline-powered vehicle.

Scope 2 emissions came from electricity (974 MTCO2e; 51%), hot water (669 MTCO2e; 35%), and chilled water (285 MTCO2e; 15%). OC does not purchase district steam as steam is produced by the on-site boiler.

Scope 3 emissions are derived from eight of the 15 categories (see Table 1). The other seven were deemed irrelevant to OC operations or contributed a *de minimis* amount (see Supplementary Table 1).

84% of Scope 3 emissions and 68% of total OC emissions resulted from three categories: Category 9: Downstream transportation and distribution (4,672 MTCO2e; 52%), which consisted solely of emissions from patient transportation to and from OC, Category 1: Purchased goods and services (1,823 MTCO2e; 20%), and Category 7: Employee commuting (1,027 MTCO2e; 12%).

In Category 9, there were approximately 200,000 patient visits to OC in FY23. The median roundtrip distance for patient travel to OC was 63 mi (IQR 32-115 mi). 98% of patients traveled from fewer than 200 mi away. Considering only Category 9 emissions, 79% came from patients living within a 100 mile drive of OC, and 40% came from patients within a 50 mile drive of OC.

In Category 1, 71% of emissions stem from the purchase of medical and surgical supplies (1,294 MTCO2e), 11% from pharmaceutical supplies (192 MTCO2e), 5% from food purchases (86 MTCO2e), and the remaining 14% from miscellaneous goods and services (252 MTCO2e), including electronics and office supplies.

Data for Category 7 came from a survey sent to OC employees with a 44% response rate. The average roundtrip commute was 42 mi with approximately 98% of commutes occurring in a personal vehicle with a combustion engine. Other identified modes of transportation included personal vehicles with an electric motor, carpools, and bicycling.

## 4 Discussion

Total emissions from OC were 11,049 MTCO2e. This is equivalent to the yearly, average emissions of 2,500 automobiles driving in the United States, and would require over 11,000 acres of mature forest to remove it from the atmosphere (17). OC emissions were allocated as follows: 2% to Scope 1, 17% to Scope 2, and 81% to Scope 3.

The distribution of OC emissions across the scopes aligns with that of a 1,233-bed Dutch hospital (8), a 1,250-bed Singaporean hospital (9), and a 2,500-bed German academic hospital (10) (see Table 3). These similarities suggest emission ratios may be generalizable across clinical centers.

**Table 3:** Ratio of total GHG emissions by scope for a US freestanding orthopedic center, a Dutch hospital, a Singaporean hospital, and a German academic hospital.

		Ratio (%)		
Facility	Location	Scope 1	Scope 2	Scope 3
Freestanding orthopedic center (OC)	US	2%	17%	81%
1,233-bed hospital	Netherlands (8)	5%	23%	72%
1,250-bed hospital	Singapore (9)	2%	16%	83%
2,500-bed academic hospital	Germany (10)	3%	26%	71%

Notably, a novel “easy-to-use approach based on financial data” applied at a 302-bed German academic hospital (11) had a markedly larger Scope 1 allocation of 37%–40%. This institution used fossil fuels directly for heating rather than electricity, which would increase Scope 1 emissions and decrease Scope 2 emissions.

### 4.1 Scope 1

The absolute value of OC's Scope 1 emissions (183 MTCO2e) are low compared to those from inpatient hospitals which ranged from 4,223 MTCO2e (9) to 9,989 MTCO2e (8). The building has just a single natural gas boiler for steam, which accounts for the majority of Scope 1 emissions (173 MTCO2e). This steam is primarily for sterile processing of procedural instruments.

The remaining Scope 1 emissions come from a single vehicle (1 MTCO2e) and anesthetic gases from the two operating rooms (9 MTCO2e). Anesthetic gases are lower than may be expected for two operating rooms for multiple reasons: (1) sevoflurane is the only volatile anesthetic used at OC and its carbon dioxide equivalent is significantly less than alternative volatile anesthetics (18), (2) many of the surgeries are performed using regional anesthesia as the primary mode of anesthesia, and (3) nitrous oxide is rarely used and only comes from e-cylinders rather than central tanks, which are known to leak (19).

Fugitive emissions from refrigerants were considered *de minimis*. The appliances in OC were recently installed and there were no records of recharges, maintenance, nor disposal of equipment containing over 50 lbs of refrigerant.

### 4.2 Scope 2

The 1,929 MTCO2e Scope 2 emissions come from electricity, hot water and chilled water. The central utility plant that services OC does not currently service other buildings, though it has the capacity to support future developments in the surrounding area. Steam is produced by an on-site boiler, rather than purchased from district utilities, so emissions from steam production fall under Scope 1. OC's electricity comes from the local grid and benefits from the renewable energy portfolio of its affiliated university. While the regional (SRVC) eGRID emission factor for OC is 0.2705 kgCO2e/kWh, OC's Scope 2 emissions utilize the university's specific emission factor for electricity (0.2435 kgCO2e/kWh) to account for the university's procurement of renewable energy.

The opportunity to reduce emissions from electricity is through either reduced consumption by emphasizing energy efficiency and conservative applications of technology or by increasing the amount of electricity generated through low-carbon or fossil fuel-free methods. In the past few decades, emission reductions from electricity generation occurred by transitioning from coal to a less carbon-intensive fossil fuel, like natural gas, and increasing fossil fuel-free options, like wind and solar (20). OC was built to accommodate rooftop solar. If a solar array was installed at OC, it could generate between 180 and 240 kWh per year and reduce Scope 2 emissions from electricity by approximately 11%.

OC's hot and chilled water are manufactured in a mostly electric utility plant, which uses a combination of heat recovery chillers, traditional chillers, and gas boilers to provide heating and cooling services. Electric heat recovery chillers efficiently capture and utilize excess heat generated in the cooling process, resulting in hot and chilled water with fewer emissions than a combination of traditional chillers and boilers. While electrification and the use of heat recovery chillers in a utility plant are generally not building-level decisions, a facility can reduce its emissions by minimizing the consumption of chilled water with lighting and temperature setbacks. These setbacks reduce the operation of lighting and temperature control features when the building is unoccupied, decreasing the need for those locations to be cooled, lowering energy consumption, and proportionally reducing energy costs.

### 4.3 Scope 3

The 8,937 MTCO2e of Scope 3 emissions was 81% of OC's total emissions. 84% of Scope 3 emissions came from three categories: Category 9 (4,672 MTCO2e; Downstream transportation and distribution), Category 1 (1,823 MTCO2e; Purchased goods and services), and Category 7 (1,027 MTCO2e; Employee commuting). Less than 1% of Scope 3 emissions came from Category 6 (1 MTCO2e; Business travel), but this is likely due to the persistence of virtual conferences as a result of travel restrictions during the COVID-19 pandemic.

The majority of Scope 3 emissions (52%) and a substantial portion of OC's total emissions (42%) came from Category 9: Downstream transportation and distribution, which accounts for patient travel to and from the clinic to receive care. Considering the overall contribution of patient travel to total OC emissions (42%), and that patient travel is not always included in hospital assessments (5, 6, 9, 11), we highlight it as an opportunity to reduce healthcare emissions through telemedicine.

#### 4.3.1 Patient travel

Two emission analyses from international hospitals included patient transportation. The Dutch hospital reported patient transportation was 3.2% of total emissions (8), while the German hospital reported patient transportation was 9% of total emissions (10). An important distinction between these hospitals and OC is the hospitals are inpatient and OC is outpatient. An outpatient center can have many different patients for each clinic room each day while an inpatient hospital has only a single patient per hospital room for multiple days. There are simply many more patients transporting themselves to and from an outpatient center than an inpatient hospital. Additionally, more resource-intensive therapies occur at inpatient hospitals (e.g., infusions, dialysis, and surgery) than at an outpatient orthopedic center (e.g., clinic visits, physical therapy, surgical follow-ups), which would proportionally make other Scope 3 categories, like Category 1: Purchased goods and services [which is 59.7% of the entire Dutch hospital's emissions (8)], higher at the inpatient hospitals than at OC.

Patients may also travel further for orthopedic consultations than other specialties as demonstrated by a retrospective review from Stanford Healthcare (21). In an attempt to ensure our Category 9 emissions assessment were not inflated by university students and part-time residents who have their mailing addresses at a location far away from their physical home in central Virginia, we capped miles driven to a 200 mile one-way trip, and assumed all addresses further than 200 mi away were residents of Charlottesville with a 0 mile distance to the clinic. Even with this conservative approach to calculating patient travel, Category 9 is a majority of the Scope 3 emissions and emits more than Scope 1 and 2 combined.

OC can reduce its emissions from patient travel by optimizing the use of telemedicine (21, 22). Orthopedic patients are open to telehealth and view it as an equivalent to in-person visits (23). Applying telemedicine in situations where it optimizes clinician workflow and patient care is an opportunity to improve multiple facets of healthcare while eliminating a large source of GHG emissions and air pollution (6).

In situations where telemedicine is not optimal for clinician workflow or patient care, a reduction in patient travel emissions could come from a reduction in patients' vehicle emissions. The installation of Level 3 electric chargers in the patient parking lot could encourage patients to drive electric vehicles to their visits by addressing range anxiety.

#### 4.3.2 Purchased goods and services

Category 1: Purchased goods and services, which includes all the supplies required to facilitate clinical, operational and administrative functions, accounted for 20.4% of OC's Scope 3 emissions. The top contributor to this category was medical supplies (1,294 MTCO2e). A Canadian study with a different methodology (statistical sampling and inference) also identified medical products as the highest contributor among goods and services (6).

OC's Category 1 emissions were 17% of total OC emissions. In comparison, purchased goods and services contributed to approximately 41% of total emissions in a hospital in Germany (10) and over 40% of total emissions in a hospital in Singapore (9). While our methodology for calculating the purchased goods and services was similar to these two studies, it is likely that different emission factors associated with national supply chains and energy production contributed to this variance. There are also differences between the institutions evaluated (i.e., inpatient vs. outpatient) and larger methodological differences, such as the Singaporean study not including patient travel (9).

Purchased goods and services, specifically medical supplies (6) and potentially pharmaceuticals (9), are significant contributors to GHG emissions. Reducing these emissions will require a more selective application of these items with a focus on reducing unnecessary waste which clinicians have voiced willingness to do (24). Alternatively, hospitals and health systems can work with vendors through contracting and advocacy to encourage reduction in the upstream and downstream energy required to create, use, and dispose of their products.

#### 4.3.3 Employee commuting

Category 7: Employee commuting was 9% of OC's total GHG emissions. Our employee transportation survey had a 44% response rate. The average employee roundtrip was 42 mi with approximately 98% commuting in a single-occupancy vehicle with a combustion engine.

Employee commuting contributed to approximately 3% of total emissions in both the Dutch (8) and German (10) hospitals. This large difference is in part associated with the frequency of fossil fuel-free bike commuting that occurs in the Netherlands (25% of daily commutes) and Germany (9% of daily commutes) compared to the United States (1% of daily commutes) (25). The university affiliated with our hospital incentivizes alternative transportation, rather than a single-occupancy vehicle, but this works best for employees who live in close proximity to OC.

In a semi-rural community with an average roundtrip commute of 42 mi, a reduction on employee transport emissions will need to focus on reducing automobile emissions. Installing Level 1 or 2 electric chargers in the employee garage could encourage employees to purchase plug-in hybrid or fully electric vehicles while providing an incentive of reduced commuting costs. For areas where on-site parking is limited, the implementation of strategic bus routes from well-placed satellite parking lots could reduce emissions from personal vehicles, while also potentially alleviating commuting challenges for employees, such as a lack of public transportation infrastructure or traffic congestion.

### 4.4 Emissions per full time equivalent

Emissions per FTE are 33.2 MTCO2e per FTE which is much greater than the average of 3.2 (maximum 7.1) MTCO2e per FTE reported in Swiss hospitals (5) or 16–23 MTCO2e per FTE reported by a Canadian hospital (6). However, an important distinction is our study included patient (4,672 MTCO2e) and clinician (1,027 MTCO2e) transportation and neither the Swiss nor the Canadian study did. Eliminating patient transportation and employee commuting from our total emissions results in 19.2 MTCO2e per FTE which is in the range of the Canadian hospital.

Macroeconomically, GHG emissions from the US health sector account for a higher percentage of national emissions at 8.5% (2) than the Swiss health sector at 6.7% (26). There is an even starker difference between national per capita GHG emissions between the two countries with the US at 17.2 MTCO2e and Switzerland at 4.5 MTCO2e (26). This difference in carbon intensity may explain why OC's GHG emissions per FTE are six times greater than hospitals in Switzerland.

### 4.5 Emissions per square meter

OC's emissions per square meter are 0.6 MTCO2e per year compared to the 0.01–0.07 MTCO2e per square meter per year in public hospitals in Punjab, Pakistan (7). After eliminating patient and clinician transportation, which the Pakistani study did not include, OC generates 0.4 MTCO2e per square meter per year, which is five to 24 times larger. Similar to the comparison with the Swiss hospital and the carbon intensity of the Swiss economy, the US economy creates nearly 8 times more GHG emissions than the Pakistani economy (27).

### 4.6 Limitations

The data collection for our analysis was completed within 18 months of OC's opening and LEED Silver certification. Total emissions may be lower than an average orthopedic clinic because the building was designed and built for electrification and efficiency. Additionally, no major clinical nor facility infrastructure was replaced during our data monitoring.

Our analysis has the limitations of all carbon footprint analyses that rely upon industry-average emission factors. Our outcomes are not representative of the individual choices of our vendors (e.g., if a vendor has eliminated Scope 1 and 2 emissions, this will not be reflected in our calculations). To obtain vendor-specific emissions data, we recommend institutions add clauses to their vendors' contracts requesting standardized emissions and pollution-related details associated with products and processes.

### 4.7 Future direction

**Figure 2:**
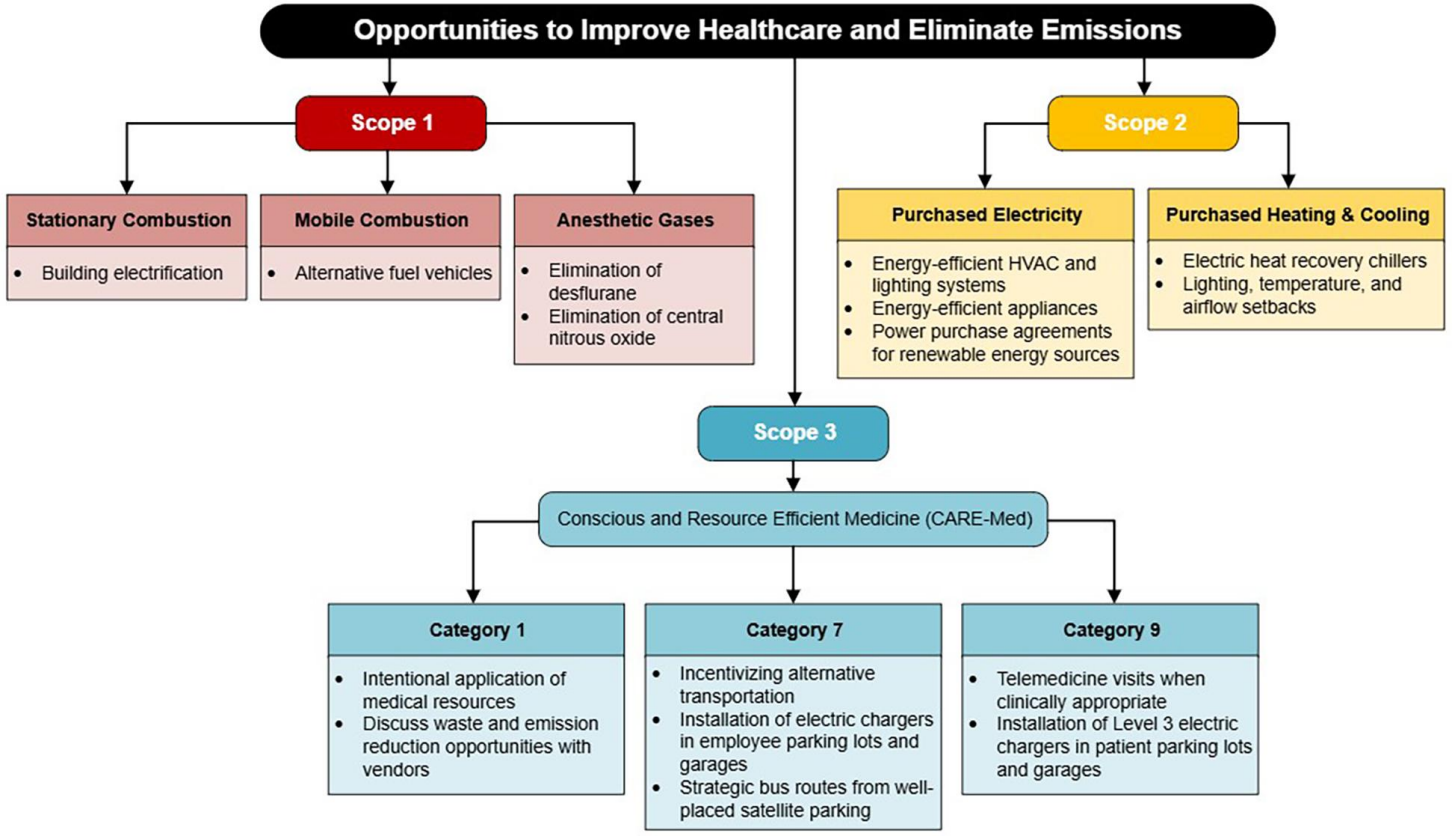
Interventions to reduce emissions from major sources by scope and category.

The principles of sustainability resonate with conventional health system strategies of high-value healthcare, patient-centered care, and evidence-based medicine. A comprehensive understanding of the impact of healthcare pollution upon patient outcomes, including delivery of care and exacerbation of health conditions, is needed. Research into healthcare and pollution must focus upon (1) the identification of the large sources of emissions, (2) categorization of those sources of emissions based upon health system potential to influence the generation of the pollution, and (3) the creation of a generalizable strategy to eliminate healthcare-associated pollution (see Figure 2). Specific to this study, we are interested to see how telemedicine can be applied more broadly in clinical orthopedics.

As one of the first peer-reviewed Scope 3-inclusive GHG inventories of a freestanding outpatient healthcare facility in the US, this study fills a critical data gap. It also offers a replicable model for health systems seeking to identify high-intensity areas to reduce their total emissions in an effort to meet net-zero targets and maintain excellent patient care.

## Data Availability

The datasets presented in this article are not readily available because the data in this study comes from multiple health system and university databases. Some of this data is associated with cost/strategic measurements and considered proprietary/privileged. Requests to access the datasets should be directed to mmeyer@virginia.edu.
