# Supplementary material for: Greenhouse gas emissions of a large, academic outpatient orthopedic center in the United States
Source: Front Health Serv. 2025 Oct 20;5:1675827. doi: 10.3389/frhs.2025.1675827 (PMC12580340; doi:10.3389/frhs.2025.1675827)
Supplement: Supplementary file 1 [file Table1.docx]

**Supplemental Table 1:** Explanation of Scope 3 categories’ relevancy to the Greenhouse Gas assessment of a freestanding orthopedic center and data required for relevant categories. FTE: full-time equivalent. FY: financial year. IACO: International Civil Aviation Organization. PGH: Practice Greenhealth. OC: orthopedic center.

|  | **Scope 3 Category** | **Relevant** | **Data Required for Relevant Categories / Rationale Category is Not Relevant** |
| --- | --- | --- | --- |
| 1 | Purchased goods and services | Y | Complete list of goods and supplies purchased by OC during FY. Items assigned to categories predefined by PGH Health Care Emissions Impact Calculator V1.3^a^, which provided emission factors. |
| 2 | Capital goods | Y | Complete list of capital goods purchased by OC during FY. Items assigned to categories predefined by PGH Health Care Emissions Impact Calculator^a^, which provided emissions factors. |
| 3 | Fuel- and energy-related activities | Y | Consumption of purchased fuels and electricity, assessed using PGH Health Care Emissions Impact Calculator.^a^ |
| 4 | Upstream transportation and distribution | Y | Suppliers for Category 1 and 2 goods and services, distance between suppliers’ distribution centers and OC, number of deliveries per year to OC, average shipment weight for each supplier. |
| 5 | Waste generated in operations | Y | Types of waste generated, the amount of waste produced per type, the waste treatment method applied to each waste stream. For recycling: volume of container, weight of cardboard bale. |
| 6 | Business travel | Y | For air travel: number of passengers, cabin class, location of departure and arrival ICAO Carbon Emissions Calculator.^b^ For lodging: spend on lodging (PGH Health Care Emissions Impact Calculator.^a^ |
| 7 | Employee commuting | Y | Mode of transportation, average commuting distance, average commuting days per year, on-site FTE. |
| 8 | Upstream leased assets | N | There are no formal agreements for the leasing of space or equipment from another entity. |
| 9 | Downstream transportation and distribution | Y | Patient travel: appointment status, postal code of patients’ addresses, distance from facility. |
|  |  | N | Transportation and distribution of sold products: on-site fabrication lab produces orthopedic devices (e.g., casts, braces, prosthetics) for patients. Given the limited quantity of devices and minimal packaging, emissions were considered *de minimis*. |
| 10 | Processing of sold products | N | The fabrication lab produces orthopedic devices (e.g., casts, braces, prosthetics) for patients. Given the limited quantity of devices and minimal packaging, emissions were considered *de minimis*. |
| 11 | Use of sold products | N | The fabrication lab produces orthopedic devices (e.g., casts, braces, prosthetics) for patients. These devices emit limited to no greenhouse gases during use, thus emissions were considered *de minimis*. |
| 12 | End-of-life treatment of sold products | N | The fabrication lab produces orthopedic devices (e.g., casts, braces, prosthetics) for patients. Given the limited quantity of devices and minimal packaging, emissions were considered *de minimis*. |
| 13 | Downstream leased assets | N | No lease agreement for space or equipment to an external entity. |
| 14 | Franchises | N | OC is neither a franchisee nor a franchisor. |
| 15 | Investments | N | The boundaries of this study cover the facility level of operations. |

Citations in Supplemental Table 1

1. Practice Greenhealth. Health Care Emissions Impact Calculator V1.3 [Internet]. Washington (DC): Practice Greenhealth; Available from:<https://practicegreenhealth.org/tools-and-resources/health-care-emissions-impact-calculator> [Accessed: June 3, 2025]
2. ICAO. ICAO Carbon Emissions Calculator. Available from: <https://www.icao.int/environmental-protection/Carbonoffset/Pages/default.aspx> [Accessed March 1, 2025]
