# Supplementary material for: Greenhouse gas emissions of a large, academic outpatient orthopedic center in the United States
Source: Front Health Serv. 2025 Oct 20;5:1675827. doi: 10.3389/frhs.2025.1675827 (PMC12580340; doi:10.3389/frhs.2025.1675827)
Supplement: Supplementary file 2 [file Table2.docx]

**Supplemental Table 2:** Explanation of data assumptions required to calculate relevant Scope 3 categories. PGH: Practice Greenhealth. OC: orthopedic center.

|  | **Scope 3 Category** | **Data Assumptions** |
| --- | --- | --- |
| 1 | Purchased goods and services | a) A small subset of purchased items were too vague in their description to be confidently allocated to a PGH category. The maximum cost of these items was $1,500 and determined to have a *de minimis* impact upon the total analysis. |
| 4 | Upstream transportation and distribution | a) For Category 1 suppliers, the top ten suppliers represent the vast majority of deliveries and emissions; emissions from other Category 1 suppliers were deemed *de minimis*.  b) For Category 2 suppliers and nine of the top ten Category 1 suppliers, any goods ordered on the same date arrived in the same shipment (the remaining Category 1 supplier delivers a shipment every day the facility is open).  c) Fully loaded box truck is six tons.^a^  d) Cargo in a fully loaded tractor trailer is 25 tons. Fully loaded tractor trailer (40 tons)^a^ ; empty tractor trailer (15 tons).  e) The typical delivery vehicle and average amount of cargo space occupied by OC’s supplies for the top ten Category 1 suppliers was provided by an on-site employee.  f) For all Category 2 suppliers, the mode of delivery was a tractor trailer and a quarter of its cargo space was occupied by the facility’s supplies.  g) Distance between the supplier and facility was estimated based on the supplier’s nearest known shipping facility, which for certain suppliers was the point of manufacturing. |
| 5 | Waste generated in operations | a) Regulated medical waste is estimated at 7% of total waste.^b^  b) One cart of mixed recyclables and one bale of cardboard is picked up each week.  c) Cardboard bale weighs 687.5 lbs (mid-point of weight range provided by cardboard baler manufacturer). |
| 6 | Business travel | a) For air travel: a direct flight - departure location is the closest airport with a direct flight to destination.  b) For air travel: economy cabin class. |
| 7 | Employee commuting | a) The survey results (44% response rate) reflect the employee population. |
| 9 | Downstream transportation and distribution | a) Passenger car is the mode of transportation.  b) Patients with addresses >200 miles from Charlottesville were assumed to be Charlottesville residents (students or part-time) with a 0 mile distance to the clinic. |

Citations in Supplemental Table 2

1. Pennsylvania Department of Transportation. Average Vehicle Weight. Available from: <https://web.archive.org/web/20240914072713/https://www.dot.state.pa.us/public/pdf/InfoBridge/Approximate%20vehicle%20weights.pdf> [Accessed: June 25, 2025]
2. Practice Greenhealth. Sustainability Benchmark Data. 2023.
